# Miller-Fisher syndrome presenting with headache and ophthalmoparesis: a case report and literature review

**DOI:** 10.3389/fmed.2025.1575696

**Published:** 2025-09-08

**Authors:** Jin Huang, Lingling Lin, Xuerong Huang, Xiaoxiao Yan

**Affiliations:** Department of Neurology, The Third Affiliated Hospital of Wenzhou Medical University, Wenzhou, China

**Keywords:** headache, ophthalmoplegia, Miller-Fisher syndrome, case report, literature review

## Abstract

Miller-Fisher syndrome (MFS) is a rare variant of Guillain-Barre syndrome, classically characterized by a triad of symptoms, including ataxia, areflexia, and ophthalmoplegia. However, only a few cases have documented clinical data on a rare atypical presentation of MFS, with headache and ophthalmoplegia as the initial manifestation. We report an 83-year-old Chinese female patient with no history of respiratory or gastrointestinal infection presented with headache and ophthalmoplegia. Cerebrospinal fluid analysis showed albuminocytological dissociation, along with positive anti-GQ1b antibodies, leading to a diagnosis of incomplete Miller-Fisher syndrome. The patient’s headache symptoms were relieved following immunoglobulin treatment, and ophthalmoplegia resolved within 20 days. A literature search identified eight cases of MFS patients initially presenting with headaches, followed by ophthalmoplegia. Detection of anti-ganglioside antibodies in serum or cerebrospinal fluid enables early diagnosis of MFS, and early immunoglobulin treatment improves patient prognosis.

## 1 Introduction

Miller-Fisher syndrome (MFS), first described by Fisher in 1956, is characterized by specific symptoms, including ataxia (lack of coordination), ophthalmoplegia (weakness of eye muscles), and areflexia (absence of tendon reflexes). It is considered a variant of Guillain-Barré syndrome (GBS) (1). Atypical MFS is characterized by a single-symptom presentation, increasing the risk of being misdiagnosed or overlooked compared with typical manifestations. The present study retrospectively analyzed the data of a patient admitted to the Third Affiliated Hospital of Wenzhou Medical University in March 2024, initially presenting with headache, followed by bilateral ophthalmoplegia. The preliminary diagnosis was painful ophthalmoplegia, followed by a diagnosis of incomplete MFS. Additionally, relevant literature reports were summarized to enhance physicians’ clinical awareness of this disease.

## 2 Case presentation

An 83-year-old Chinese female patient with a history of diabetes and no previous history of recurrent headaches visited the Endocrinology Department of the Third Affiliated Hospital of Wenzhou Medical University on March 26, 2024. On admission, blood glucose monitoring indicated that the patient’s levels were well-controlled, with no instances of hypoglycemia or hyperglycemia. She reported experiencing recurrent headaches and a decreased appetite for 10 days prior, without identifiable triggers. The headache began as a mild throbbing in both temples but gradually intensified into a sensation of fullness in the eye sockets, accompanied by a reduced appetite. She frequently woke up at night due to pain but reported no recurrent nausea or vomiting. Nonsteroidal antipyretic analgesics were prescribed for pain relief, but they did not significantly alleviate the headache. Due to the patient’s headache, we carefully inquired about any recent upper respiratory tract or intestinal infections and tested for infectious disease pathogens, including campylobacter and arboviruses, as well as other neurotropic viruses such as herpes and CMV, none of which were positive. On March 29, 2024, the patient experienced bilateral ptosis, characterized by drooping of the eyelids and accompanied by blurred vision. An ophthalmology consultation was sought, during which bilateral eye pressure and fundus examinations were conducted, revealing no notable abnormalities. Consequently, a referral to our department was made to explore the potential diagnosis of painful ophthalmoplegia. Upon admission, neurological examination revealed a blood pressure of 129/80 mm Hg, with the patient exhibiting clear consciousness and fluent speech. No irregularities were detected in the heart, lungs, or abdominal region. The pupils were uniform and circular, measuring approximately 5.0 mm, with no light reflex detected. Both eyes exhibited limited extraocular movements, with the gaze fixed in the primary position. No sensory impairment was noted, and other cranial nerves appeared normal. Muscle strength in all extremities was rated at 5, muscle tone was normal, and bilateral tendon reflexes were absent. Coordination of voluntary movements across all limbs was normal, with no noticeable irregularities in either deep or superficial sensation. Laboratory tests, including complete blood count, C-reactive protein, liver and kidney function assessments, thyroid hormones and related antibodies, autoimmune antibodies (ANA), vasculitis markers, screenings for HIV, syphilis, and tumor markers [neuron-specific enolase (NSE), cancer antigen 125 (CA125), CA199, CA153, alpha-fetoprotein (AFP), squamous cell carcinoma antigen (SCC), were all within normal ranges. Imaging examinations, including head and neck computed tomography angiography (CTA)], did not indicate the presence of any arterial aneurysms. Additionally, both plain and contrast-enhanced magnetic resonance imaging (MRI) scans of the cranial and orbital regions revealed no abnormalities. Considering the patient’s advanced age, we performed a chest CT, an abdominal CT, and a gynecological ultrasound to rule out the presence of tumors. No significant abnormalities were identified.

Lumbar puncture (performed 15 days into the course): The analysis of the cerebrospinal fluid (CSF) examination revealed a colorless and transparent appearance, with a pressure of 120 mm H_2_O. White and red blood cell counts were 1 cell/μL, and Pandy’s test was weakly positive. The glucose concentration was 4.05 mmol/L, protein quantification was 67.3 mg/dL, and the chloride concentration was 125.4 mmol/L. Electromyography revealed multiple peripheral nerve injuries, including myelin damage in both motor and sensory nerves, as well as axonal changes. The serum test for anti-GQ1b antibody was positive, while tests for anti-GT1b antibody, anti-GD1b antibody, anti-GD1a antibody, and anti-sulfatide antibody were negative.

A clinical diagnosis of incomplete Miller Fisher syndrome (MFS) was established based on the protein-cell dissociation in the cerebrospinal fluid (CSF) and the presence of positive serum anti-GQ1b antibodies. The patient received intravenous immunoglobulin (IVIG) at a dose of 0.4 g/kg/day for 5 days, in conjunction with mecobalamin and thioctic acid (a-lipoic acid) injections. After the 5-day course of intravenous immunoglobulin therapy, the headache symptoms diminished, and the bilateral pupil light reflexes exhibited improved reactivity; nevertheless, bilateral ophthalmoplegia continued. After two weeks, clinical signs in both eyes had improved, and there was a partial recovery of extraocular motility, with complete resolution of the bilateral ophthalmoplegia deficit seven weeks after onset.

## 3 Discussion

MFS is an immune-mediated inflammatory demyelinating disorder of the peripheral nerves, characterized by clinical symptoms including total external ophthalmoplegia, ataxia, and areflexia. It is a clinically rare condition, with an incidence rate of 1–2 per 100,000 (2). MFS constitutes 15–25% of Guillain-Barré Syndrome (GBS) cases in Asia, in contrast to 1–7% in Western countries (2), suggesting a higher prevalence among Asian populations. The cause of this disparity is not clearly understood, although environmental factors may contribute to it (3).

Similar to GBS, MFS typically appears a few days to weeks after infections caused by pathogens like *Campylobacter jejuni*, Cytomegalovirus, and Epstein–Barr virus. Molecular mimicry is recognized as the primary mechanism driving the disease. Over 90% of MFS patients have elevated serum anti-ganglioside antibody levels, specifically anti-GQ1b antibodies (4). According to the 2014 classification and diagnostic criteria for Guillain-Barré syndrome (GBS) and Miller Fisher syndrome (MFS), these conditions, along with Bickerstaff’s brainstem encephalitis, are considered part of a continuous disease spectrum, categorized based on clinical presentation (5). MFS is categorized into incomplete and central nervous system (CNS) subtypes. The incomplete subtype is further classified based on the presence or absence of three key symptoms: ophthalmoplegia, ataxia, and ptosis, as well as pupillary dilation (5).

Our patient initially presented with headaches, followed by ophthalmoplegia, ptosis, and pupillary dilation, but without ataxia — a rare clinical presentation. Although the patient had a history of diabetes, the clinical manifestations indicated involvement of multiple cranial nerves, which contrasted with the typical single nerve involvement characteristic of diabetic cranial neuropathy, thereby ruling it out. The onset of severe headaches, followed by ophthalmoplegia, prompted the initial consideration of painful ophthalmoplegia. Ultimately, the presence of positive anti—GQ1b antibodies in the serum confirmed a diagnosis of incomplete Miller Fisher syndrome (MFS).

Subsequently, a systematic search was conducted in “PubMed,” “Web of Science,” and “Cochrane” using the keywords “Headache,” “Ophthalmoplegia,” “Miller Fisher syndrome,” or “MFS” from January 1, 2010 to January 1, 2024 to provide a comprehensive overview of the clinical features and prognostic information related to the association between “Headache,” “Ophthalmoplegia,” and “Miller Fisher syndrome.” An additional seven patients identified in English-language articles (6–12) were included in our descriptive analysis (Table 1). The median age of the cohort, which included three men and five women, was 37 years (ranging from 9 to 83 years). Among the eight patients, one had enteritis before the onset of symptoms, three experienced upper respiratory tract infections, one had a fever, and two reported no previous illnesses. All patients tested positive for immunoglobulin G (IgG) anti-GQ1b antibody, indicating its role as a diagnostic marker for MFS. Additionally, two cases tested positive for IgG anti-GT1a antibodies.

**Table 1:** Demographic, clinical, and laboratory characteristics of the 8 patients.

Number	Age/sex	Antecedent infection	Main clinical manifestations	Antiganglioside antibody	Treatment	Recovery time
1	9/F	Fever	Headache, ophthalmoplegia, epilepsy and dyspepsia	anti-GQ1b IgG	IVIG followed by plasmapheresis	2 months
2	41/M	Respiratory tract infection	Headache, ophthalmoplegia	anti-GQ1b IgG	Not specified	5 months
3	25/M	Diarrheal	Headache, ophthalmoplegia, dizziness	anti-GQ1b IgG	IVIG	1 week
4	49/F	None	Headache, ophthalmoplegia	anti-GQ1b IgG, anti-GT1a IgG	IVIG	1 week
5	53/F	Respiratory tract infection	Dysarthria, dysphagia, diplopia, headache, ophthalmoplegia	anti-GQ1b IgG, anti-GT1a IgG	IVIG	3 months
6	11/M	Fever	Headache, dizziness, vomiting, diplopia, ataxia, ophthalmoplegia	anti-GQ1bIgG	IVIG	2 months
7	26/F	Respiratory tract infection	Headache, ophthalmoplegia	anti-GQ1bIgG	IVIG	2.5 months
8 (our case)	83/F	None	Headache, ophthalmoplegia	anti-GQ1bIgG	IVIG	0.5 month

The eight cases displayed an acute onset, primarily characterized by headaches and ophthalmoplegia. Among these, six patients had a history of previous infections, whereas two cases did not present a definitive trigger. The patient experienced headaches and diplopia, but neither ataxia nor absent tendon reflexes were present. The initial diagnosis was painful ophthalmoplegia, and steroid therapy was initiated. However, there was no clinical improvement, and the MRI did not reveal any inflammatory changes in the cavernous sinus. Ultimately, serum analysis revealed the presence of positive GQ1b antibodies, prompting consideration of Miller Fisher Syndrome (MFS). Consequently, treatment was transitioned to intravenous immunoglobulin (IVIG), which significantly improved extraocular motility. Notably, Cases 2, 4, and 7 also did not display ataxia, which could easily lead to misdiagnosis. Consequently, clinicians must remain vigilant with these cases, as the diagnosis primarily relies on the detection of positive anti-GQ1b IgG antibodies in the serum. Research indicates that the positive rate of GQ1b antibodies in MFS patients can be as high as 83% (13), with a rate of 81% observed within the first week (14). However, the positive rate for anti-GQ1b antibodies is lower in cases of incomplete MFS. A study revealed that the positive rate for anti-GQ1b antibodies in patients with acute isolated abducens nerve palsy was only 25%, possibly due to the limited extent of the lesion and milder symptoms, resulting in lower antibody levels (15).

Case 5, with a history of an upper respiratory tract infection, experienced an acute onset. The patient presented with a headache, diplopia, and weakness in the muscles of the mouth and throat. Brainstem encephalitis (Bickerstaff brainstem encephalitis, BBE) was initially suspected; however, MRI results showed no significant abnormalities. A physical examination revealed absent limb reflexes accompanied by ataxia, while electromyography indicated peripheral neuropathy. The patient was diagnosed with a pharyngeal-cervical-brachial (PCB) variant of GBS. The PCB variant of GBS is a rare, acquired peripheral neuropathy characterized by acute paralysis of the muscles in the mouth, neck, and upper limbs, with reduced reflexes in the upper limbs and minimal or no lower limb involvement (16). Both the PCB variant of GBS and MFS fall within the same disease spectrum and may exhibit overlapping features. Clinical symptoms can include facial nerve paralysis, sensory impairment, ataxia, ophthalmoplegia, ptosis, and so forth, with the most common overlap occurring with MFS (17–19). Approximately 60% of patients with acute Guillain-Barré syndrome (GBS) may have detectable anti-ganglioside antibodies in their blood, with variations in ganglion-specific antibodies correlating with different subtypes of GBS (20). MFS is associated with GQ1b and GT1a, whereas the PCB variant of GBS is associated with GT1a, consistent with the findings in case 5 (21). Clinicians should therefore pay close attention to bulbar symptoms and conduct thorough examinations to identify potential overlaps as early as possible.

The GQ1b antibody is a key biological marker for MFS. The GQ1b antigen is highly expressed in the oculomotor, trochlear, and abducens nerve, as well as in the muscle spindles of the limbs and the reticular structure of the brainstem (22). Microbes carrying the GQ1b antigenic determinant cluster can induce the production of anti-GQ1b antibodies, which bind to the GQ1b antigen in the oculomotor, trochlear, and abducens nerve, leading to ophthalmoplegia (22). Patients with acute ophthalmoplegia should not have Miller Fisher Syndrome (MFS) dismissed solely on the basis of a single lumbar puncture cerebrospinal fluid (CSF) result. Instead, the diagnosis should be confirmed through a comprehensive physical examination, additional relevant factors, and the consideration of a single lumbar puncture result.

Furthermore, this cohort of patients experienced headaches, a less common symptom of MFS. Jung et al. (23) reported that 6 out of 38 MFS patients (16%) had headaches. Koga et al. (24) found that 22% of patients experienced pain during the acute phase of MFS, of whom 50% reported pain around the eye socket. In contrast, pain in the back and extremities is notably widespread. The two most prevalent types of pain among various categories are muscular pain and neuropathic pain.

The etiology of headaches remains uncertain; however, several potential mechanisms have been proposed: (1) Increased intracranial pressure can occur due to obstruction of the subarachnoid space from elevated protein levels in the CSF (25). The patient did not display symptoms commonly linked to intracranial hypertension, such as nausea or vomiting, and the cerebrospinal fluid (CSF) pressure was normal, which does not account for the patient’s headache. (2) Since the description of Posterior Reversible Encephalopathy Syndrome (PRES) in 1996, numerous case reports have documented the simultaneous concurrent occurrence of PRES and acute GBS. However, the mechanism underlying the coexistence of PRES and GBS remains unclear. A significant hypothesis suggests that autonomic dysfunction-induced hypertension exceeds the cerebrovascular regulatory threshold, leading to vasogenic edema (26). On the other hand, a high prevalence of autoimmune disorders has been identified as risk factors in PRES. As reported by Pilato and colleagues, the presence of systemic immune impairment should be considered in the pathogenesis of PRES, particularly in normotensive patients (27). Moreover, the hypothesis of altered endothelial function has been proposed, particularly in patients with autoimmune diseases (28). Additionally, the release of several cytokines in GBS (tumor necrosis factor-*α*, interleukin-6, interferon-*γ*, and IL-17) (29) responsible for systemic immune activation, which, in our hypothesis, could lead to endothelial dysfunction and altered vascular permeability, is seen in PRES (3). The role of antibody-mediated trigeminal vascular pain pathways has also been investigated. Friedman et al. (30) found that GD3 and GD1b antigens are present in all cranial nerves and the spinal cord. Antibodies against gangliosides may cause demyelination of spinal and cranial sensory nerves, potentially activating trigeminal vascular pain pathways, which may result in headaches. Although patients in the present study tested negative for anti-GD3 and anti-GD1b antibodies, Kaida et al. (31) noted that GQ1b and GT1a antigens are sparsely distributed in other cranial nerves, such as the trigeminal nerve. Therefore, despite the negative results for anti-GD3 and anti-GD1b antibodies, it is still plausible that a minor presence of GQ1b antigen within cranial nerves, including the trigeminal nerve, could have contributed to the headaches experienced by the patients. Our patient suffered from severe headaches that did not respond to anti-inflammatory or analgesic medications, including duloxetine or pregabalin. Nonetheless, significant relief was achieved following IVIG, which supports the hypothesis of antibody-mediated activation of trigeminal vascular pain pathways.

Currently, there is ongoing discussion regarding the management of MFS. The condition typically exhibits a self-limiting trajectory, with symptoms often resolving spontaneously. Nevertheless, IVIG and plasma exchange can improve recovery from ophthalmoplegia and ataxia, potentially shortening the illness. The current study found that seven patients receiving gamma globulin therapy exhibited significant alleviation of symptoms.

## 4 Conclusion

Patients with acute headache onset, accompanied by ophthalmoplegia, elevated CSF protein levels, and increased serum anti-GQ1b antibody levels, should be evaluated for incomplete MFS. Incomplete MFS typically has a positive prognosis, and the decision to initiate immunomodulatory therapy should be guided by the patient’s overall condition.

## Data Availability

The original contributions presented in the study are included in the article/supplementary material, further inquiries can be directed to the corresponding authors.
